# Englerin A Agonizes the TRPC4/C5 Cation Channels to Inhibit Tumor Cell Line Proliferation

**DOI:** 10.1371/journal.pone.0127498

**Published:** 2015-06-22

**Authors:** Cheryl Carson, Pichai Raman, Jennifer Tullai, Lei Xu, Martin Henault, Emily Thomas, Sarita Yeola, Jianmin Lao, Mark McPate, J. Martin Verkuyl, George Marsh, Jason Sarber, Adam Amaral, Scott Bailey, Danuta Lubicka, Helen Pham, Nicolette Miranda, Jian Ding, Hai-Ming Tang, Haisong Ju, Pamela Tranter, Nan Ji, Philipp Krastel, Rishi K. Jain, Andrew M. Schumacher, Joseph J. Loureiro, Elizabeth George, Giuliano Berellini, Nathan T. Ross, Simon M. Bushell, Gül Erdemli, Jonathan M. Solomon

**Affiliations:** 1 Novartis Institutes for Biomedical Research, Cambridge, Massachusetts, United States of America; 2 Novartis Institutes for Biomedical Research, East Hanover, New Jersey, United States of America; 3 Novartis Institutes for Biomedical Research, Horsham, United Kingdom; 4 Novartis Institutes for Biomedical Research, Basel, Switzerland; 5 Genomics Institute of the Novartis Research Foundation, San Diego, California, United States of America; University of Houston, UNITED STATES

## Abstract

Englerin A is a structurally unique natural product reported to selectively inhibit growth of renal cell carcinoma cell lines. A large scale phenotypic cell profiling experiment (CLiP) of englerin A on ¬over 500 well characterized cancer cell lines showed that englerin A inhibits growth of a subset of tumor cell lines from many lineages, not just renal cell carcinomas. Expression of the TRPC4 cation channel was the cell line feature that best correlated with sensitivity to englerin A, suggesting the hypothesis that TRPC4 is the efficacy target for englerin A. Genetic experiments demonstrate that TRPC4 expression is both necessary and sufficient for englerin A induced growth inhibition. Englerin A induces calcium influx and membrane depolarization in cells expressing high levels of TRPC4 or its close ortholog TRPC5. Electrophysiology experiments confirmed that englerin A is a TRPC4 agonist. Both the englerin A induced current and the englerin A induced growth inhibition can be blocked by the TRPC4/C5 inhibitor ML204. These experiments confirm that activation of TRPC4/C5 channels inhibits tumor cell line proliferation and confirms the TRPC4 target hypothesis generated by the cell line profiling. In selectivity assays englerin A weakly inhibits TRPA1, TRPV3/V4, and TRPM8 which suggests that englerin A may bind a common feature of TRP ion channels. *In vivo* experiments show that englerin A is lethal in rodents near doses needed to activate the TRPC4 channel. This toxicity suggests that englerin A itself is probably unsuitable for further drug development. However, since englerin A can be synthesized in the laboratory, it may be a useful chemical starting point to identify novel modulators of other TRP family channels.

## Introduction

**Fig 1:**
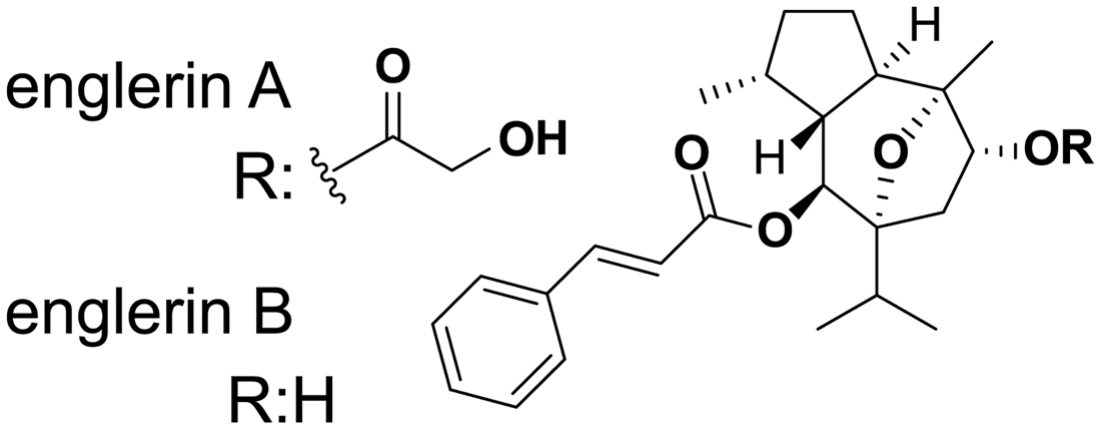
Chemical structures of englerin A and englerin B.

Natural products are secondary metabolites most commonly isolated from plants and microorganisms. Bioactive natural products are highly evolved chemical species which often bind tightly to their targets to exert their biological activity and have been a rich source of new pharmaceutical compounds[1, 2]. The guaiane sesquiterpene englerin A (Fig 1a) was isolated in 2008 from the bark of the African plant *phyllanthus engleri* [3]. Its unique chemical structure suggested to us and others[4] that englerin A may bind a novel target. Englerin A is of medicinal interest because it preferentially inhibits growth of renal cell carcinoma (RCC) cell lines in the NCI-60 panel[3] and preferentially inhibits RCC cell line growth relative to gliobastoma, breast, prostate, and non-transformed kidney cells[5, 6]. Englerin A is also attractive to medicinal chemists because it can be synthesized in the laboratory[4, 7].

There has not been a consensus on the mechanism by which englerin A inhibits RCC cell growth. Englerin A directly activated protein kinase C (PKC) theta in a biochemical assay and was hypothesized to affect cell viability by promoting glucose dependence while simultaneously starving cells of glucose[6]. Others observed that englerin A caused necrosis, an increase in reactive oxygen species and an influx of calcium into RCC cells[5]. In the A-498 RCC cell line necrosis and apoptosis, a G2/M cell cycle block, induction of autophagy, and inhibition of Akt and Erk kinases were observed[8].

In this report, compound profiling in cellular assays was used to approach the often difficult challenge of compound target identification[9–12]. The strategy was to run cell proliferation assays on a large panel (>500) of well characterized cell lines from the cancer cell line encyclopedia[13]. We examined the cell line features which best correlated with resistance and sensitivity to englerin A as a novel approach to determine its mechanism of action. The data suggested the hypothesis that englerin A inhibits tumor cell line growth by activating the transient receptor potential cation channel, subfamily C, member 4 (TRPC4) ion channel. A recent paper published while our manuscript was being prepared, agrees with the efficacy target hypothesis generated by our studies[14].

## Materials and Methods

### Englerin A and englerin B sourcing

Englerin A (CAS# 1094250-15-3) was purchased from AppliChem (Missouri, USA), and CFM Oskar Tropitzsch (Marktredwitz, Germany). Englerin B was synthesized from englerin A according to the published procedure[15].

### Cell line profiling (CLiP) experiment and bioinformatics analysis

Compound profiling experiment and data analysis was performed as previously described [13]. Data was imported and plotted using the Tibco Spotfire DXP application. Lines were categorized as sensitive, intermediate and refractory as follows: Amax<-40 & Inflection Point <2 ~ **Sensitive**, Amax>-20 & Inflection Point >10 ~ **Refractory**, all else were called **Intermediate**. Cancer Cell line HG-U133 Plus 2 data was processed and normalized as described[13]. Refractory and sensitive cell lines to englerin A were compared and analyzed using the limma package within the R/Bioconductor framework. Five sets of refractory cell lines were generated from the pool of refractory cell lines trying to match the lineage composition of the set of sensitive cell lines. The limma package was used as previously described to perform the statistical comparison. Probesets of interest were significantly associated (Benjamani-Hochberg adjusted p-value < .05 and Log Fold Change > 1) in all five comparisons.

Gene expression data for the cell lines tested is available at NCBI GEO accession GSE36133, Data on the effects of englerin A and englerin B on cell line proliferation data is in S1 Table.

### Cell lines and cell culture

HEK293T, A-498, and A-673 cell lines were obtained from the Novartis cell line encyclopedia collection [13]. They were purchased from ATCC: HEK293T (Cat # CRL-11268), A-498 (Cat # HTB-44), and A-673 (Cat# CRL-1598). The HEK293T cell line with Dox-inducible expression of TRPC4 was constructed using the Invitrogen pcDNA5/FRT/TO TOPO TA expression kit (K6020-20). Human TRPC4 was ordered from Origene (Origene; RC218941). Forward primer AACATGGCTCAGTTCTATTACAAAAG and reverse primer TTAAACCTTATCGTCGTCATCC were used to PCR amplify the Myc and DDK tagged TRPC4 and the fragment was cloned into the pcRNA5/FRT/TO TOPO TA expression vector. The plasmid was cotransfected with pOG44 into the Flp-In T-REx-293 cell line, and transfected cells were selected in medium containing 100 μg/ml Hygromycin. Growth medium for HEK293T and A-673 cell lines was Dulbecco’s Modified Eagle Medium with 10% Fetal Bovine Serum (FBS), and 1% penicillin and streptomycin (P/S). Growth medium for A-498 cells was Eagles Minimum Essential Medium (EMEM) + 10% FBS with 1% P/S.

### TRPC4 siRNA rescue experiment

A-498 cells were plated at 2,000 cells/well and A-673 cells were plated at 4,000 cells/well in 96 well plates and incubated overnight at 37°C. siRNAs were transfected using RNAiMAX/OptiMEM according to the manufacturer’s protocol so that the final concentration of siRNA was 5.8 nM or 5.0 nM respectively. Plates were incubated at 37°C for 24 hours. Decreasing concentrations of englerin A (2500–0.15nM) were added to the cells. Cell viability was measured 48 hours later by Cell Titer-Glo (Promega, Madison WI). Luminescence was measured using the Perkin Elmer EnVision reader (LUM program; 200ms).

Quantitation of TRPC4 mRNA levels: RNA was isolated according to Qiagen’s RNeasy 96 kit protocol. cDNA was generated using Applied Biosystems High Capacity cDNA Reverse Transcription Kit. Quantitative PCR reactions were generated following Applied Biosystems Taqman Fast Advanced Master Mix protocol in the 384 well format with the following probes: Hu PPIA: 4333763 and Hu TRPC4: Hs01077392_m1. siRNAs used: Control: Dharmacon SmartPool Non-targeting (#D-001810-10), TRPC4: Dharmacon TRPC4-1 (*#*L-006510-10); Dharmacon TRPC4-2 (*#*L-006510-11).

### TRPC4 overexpression in HEK293T cells by transient transfection

HEK293T cells were split one day prior to electroporation. The following day cells were trypsinized and washed one time in MaxCyte electroporation buffer. Cells were resuspended in MaxCyte buffer at a density of 1×10^8^ cells/mL. Plasmid DNA was prepared at concentrations ranging from 0–300 μg/mL. 1×10^7^cells were added to each plasmid preparation and each mixture was transferred to an individual OC-100 processing assembly. The transfection was conducted on the MaxCyte STX using the “HEK293” preset protocol. The transfected cell solution was transferred to one well of a 96-well plate and incubated for 20 minutes at 37°C. The cells were then plated in a flask with 30 mL of complete growth medium. After 16 hours, cells were trypsinized and 1000 cells/well plated in a 1536 well plate for 8 hours. Each transfected cell line was treated with increasing concentrations of englerin A (0.1nM to 5000nM) for 24 hours. Cell viability was measured using Cell Titer Glo.

### Viability assay on HEK293T cells with Dox-inducible TRPC4 expression--

HEK293T cells with doxycycline inducible TRPC4 were plated at 8,000 cells/well into 96 well plates in growth medium with or without 100 ng/ml doxycycline. Four hours later, decreasing doses of 20x concentrated englerin A in growth medium were added to cells. Cell viability was measured by CellTiter-Glo 72 hours later.

#### Western blotting protocol

HEK293T cells with doxycycline inducible TRPC4 were plated in 6 well plates at a density of 500,000 cells/well and incubated at 37°C degrees for 24 hours. TRPC4 was induced by 100 ng/ml doxycycline for an additional 24 hours. Cells were lysed in lysis buffer (50mM Tris pH7.6, 150mM NaCl, 1%Triton 100, 10mM NaF, 1mM Na_3_VO_4_, 1mM EDTA, 1mM DTT) and lysates were loaded onto 4–12% Novex Bis-Tris gels. Proteins were transferred onto 0.2 μm nitrocellulose membranes. TRPC4 protein was detected using a 1:500 dilution of mouse monoclonal antibody against DDK tag (Origene, TA50011) in phosphate buffered saline (PBS) buffer containing 10% nonfat milk and 0.05% Tween-20. Antibody was then detected by ECL (Pierce/Thermo Scientific).

### PKCtheta inhibitor experiment

A-498 cells were plated at 500 cells/well and A-673 cells were plated at 20,000 cells/well in 384 well plates and incubated at 37°C for 24 hours. Decreasing concentrations of englerin A (2500–0.075nM) were added to the cells. Compound 27 obtained from the Novartis archive was added for a final concentration of 2.5μM. Cell viability was measured 48 hours later by Cell Titer-Glo.

### Viability experiment with HEK293T cells transfected with TRPC5

HEK293T cells were plated at 4,000 cells/well into 96 well plates and incubated overnight at 37°C. Cells were transfected with TRPC5 (Origene, RC213238) using FuGENE HD according to the manufacturer’s protocol. After 24 hours, decreasing doses of 20x concentrated englerin A in growth medium was added to cells. Cell viability was measured 48 hours later by Cell Titer-Glo.

### Calcium flux and membrane depolarization assays

HEK293T cells were plated at 4,000 cells/well onto clear bottom 384 well black plates and incubated for 24 hours at 37°C. Cells were transfected with TRPC expressing plasmids (Origene: TRPC4beta, RC226709, TRPC6, RC210783, TRPC4 & 5 as above) using Fugene 6 according to the manufacturer’s protocol. 72 hours after plasmid transfection, cell culture medium was removed and replaced with 20 μl of assay buffer containing 5μM probenecid. 25μl of calcium 6 or membrane potential dye was added to cells and incubated at 37°C for 2 hours or 30 minutes respectively (Assay kit: Calcium 6 assay kit, Cat. R8190; Membrane potential assay kit, Cat. R8126. Molecular Devices Inc.). Englerin A was added to cells and fluorescence was measured for three minutes using an FDSS 7000EX by HAMAMATSU with the excitation and emission wave lengths set at 480nM and 540nM. Each dose was done in duplicate. Each value was calculated by the difference of highest and lowest points of the curve, then normalized by baseline value. Dose response curves were plotted using Spotfire program.

### Electrophysiology experiments

TRPC4 currents were recorded from HEK293T cells with Dox-inducible TRPC4 on the QPatch-HT automated patch clamp system at room temperature (21–24°C) (Biolin Scientific, Stockholm, Sweden). The internal solution contained (in mM): 150 Cs-aspartate, 2 MgCl_2_, 0.36 CaCl_2_, 1 EGTA, 4 MgATP, 0.3 NaGTP and 10 HEPES, pH = 7.20 with CsOH (calculated free [Ca^2+^] = 100 nM using WebMaxChelator http://www.stanford.edu/~cpatton/maxc.html, assuming an ionic strength of 0.16). The standard external solution contained (in mM): 150 NaCl, 4 KCl, 2 CaCl_2_, 1 MgCl_2_, 10 HEPES and 10 D-glucose (pH = 7.40) with NaOH. Currents were elicited by a voltage ramp protocol consisting of a 50 ms step to −100 mV, followed by a 200 ms ramp to +100 mV and 10 ms at +100 mV. Voltage ramps were applied every 10 s from a holding potential of 0 mV. Results were expressed as means ± S.E.M.

### ML204 rescue experiment

A-673 were plated at 20,000 cells/well in 384 well plates and incubated overnight at 37°C. Englerin A was added at a final concentration of 50 nM. ML204 was added at a final concentration of 50 μM. Cell viability was measured 48 hours later using Cell Titer-Glo.

### Channel activity assays

TRP channel assays were performed as previously described [16, 17]. Non-TRP channel assays were performed as previously described [18].

### Plasma stability assay

Englerin A was incubated at 37°C in C57BL/6 mice, Sprague Dawley rats, Beagle dogs, and human plasma for 4.5 hours. 400 μl of the stock 2.5 μg/ml plasma were spiked in 10 ng/ml verapamil (1000 ng/ml verapamil spiking solution in 50:50 DMSO:water, final organic <1%). At 0, 0.5, 1.5, 2.5 and 4.5 hours, 25 μl plasma aliquots were transferred in a 96-well plate with 150 μl of acetonitrile containing 100 ng/ml of glyburide (internal standard (IS)), and centrifuged at 4,000 rpm for 10 minutes. The supernatant was transferred to a new 96-well analysis plate together with 150 μl deionized water before englerin A and B levels were determined by LC-MS/MS based on the same multiple reaction monitoring (MRM) used for the analysis of the blood samples described below. Verapamil and benfluorex were used as positive and negative control, respectively. The percentage of remaining englerin A and the formed englerin B was calculated as the analyte peak area ratio at each time divided by the analyte peak area ratio at 0 hours. Analyte peak area ratio is calculated using verapamil as the IS, while verapamil peak area ratio is calculated using glyburide as the IS.

### Quantification of englerin A and englerin B

A 25 μl aliquot of sample was subjected to protein precipitation using 150 μl of acetonitrile containing 100 ng/ml of internal standard (glyburide). After vortex and centrifugation for 5 minutes at 4000 revolutions per minute, 125 μl of supernatant was transferred to a 1 ml 96-well plate, followed by the addition of 50 μl of water. The analysis was conducted by using HPLC separation coupled with mass spectrometric detection.

Agilent 1100 (Santa Clara, CA) was used for LC separations coupled with CTC PAL (Zwingen, Switzerland). The chromatographic separation of analytes was achieved on an ACE C18 column (3 μm, 2.1 × 30 mm) from MAC-MOD Analytical, Inc. (Chadds Ford, PA), in conjunction with rapid gradient conditions and mobile phases A (water containing 0.1% formic acid) and B (acetonitrile containing 0.1% formic acid). A Sciex API 4000 Q Trap mass spectrometer equipped with a Turbo Ionspray interface from Applied Biosystems (Framingham, MA) was used for detection. The instrument was operated in the positive ion MRM mode employing nitrogen as a collision gas. The following MRM transitions were monitored: m/z 443.24 → 219.28 for englerin A, m/z 385.16 → 131.0 for englerin B and m/z 494.15 → 169.2 for internal standard, respectively. Data were acquired and processed by Sciex Analyst 1.5.2 software.

Standard regression and back-calculation of unknown concentrations were performed by Thermo Watson 7.4 software purchased from Thermo Fisher Scientific, Inc. (Philadelphia, PA). Quantification of the parent compound in rat blood was based on at least 5-point calibration curve. The assay calibration standard curve range was set from 0.1 ng/ml to 5,000 ng/ml with added 0.250 ml aliquot of pre chilled 90% Acetonitrile:10% Water mixture for protein precipitation. The bias of all calibration standards and quality control samples was within the acceptance criteria of ± 30%.

### Formulation of englerin A/B

Both englerin A and englerin B were formulated as a clear solutions in 5% ethanol, 10% polyethylene glycol 300, 5% cremophor EL, and 80% PBS. This formulation was prepared according to the Novartis approved *standard acceptable* vehicle guidance for all in-life pre-clinical evaluations.

Ethanol (200 proof ≥99.5%) was purchased from Sigma Aldrich, polyethylene glycol 300 NF was purchased from Croda, cremophore EL (now renamed to: kolliphor EL was purchased from BASF Pharma Solutions, and 10X PBS was purchased from Calbiochem. Cremophore EL was prepared as 20% (w/v) solution in deionized water and 10X phosphate buffer saline stock solution was diluted to 1X PBS prior to use in the formulation preparation.

Englerin A and englerin B were initially dissolved in ethanol and polyethylene glycol 300 with 5–10 min of water batch sonication. Sonication was applied to ensure that all powder was completely dissolved prior to addition of the cremophor EL and PBS.

Test formulations were filtered through a 0.22 μm PVDF syringe filter purchased from Merck Millipore Ltd. before *in vivo* evaluation.

### Englerin A tolerability experiments in nude mice

Tolerability studies were conducted with female athymic nude mice (Taconic, Germantown, NY) at age 8 weeks. After a single dose (subcutaneous, intra-peritoneal or oral) of englerin A, body weight and a clinical score (1 lowest to 5 highest) were determined at several time points up to 96 hours. Criteria for euthanasia were a clinical score of 2 or lower or body weight loss greater than 20%.

### Englerin A pharmacokinetic experiments

Two male Sprague Dawley rats were orally dosed with 5 mg/kg of englerin A. Blood samples (50 μl) were taken from a jugular vein catheter at 15, 30, 60, 120, 240 and 420 minutes following dosing and immediately crashed out with 250 μl of 90% ACN/ 10% water solvent mixture into polypropylene storage tubes and kept frozen approximately at -20°C until englerin A and B were quantified by LC-MS/MS as described above. One male Sprague Dawley rat was intravenously dosed with 1 mg/kg of englerin A, but died before taking the first sample at 5 minutes after dosing.

### Anesthetized rat experiments

Rats were anesthetized via an intra-peritoneal (ip) injection of ketamine/xylazine (80/10 mg/kg). A half dose (40 mg/kg ketamine) was given as supplemental anesthesia as needed. Animals were placed on a heating pad set to maintain body temperature at approximately 37°C. Body temperature was monitored throughout the experiment via a rectal temperature probe.

Once consciousness had been lost, the trachea was isolated and an endotracheal tube was inserted for mechanical ventilation. The endotracheal tube was connected to a mechanical ventilator that provided positive-pressure ventilation with room air, set to maintain 60 breaths/min with a tidal volume of 15 ml/kg. An incision was made on both sides of the neck. Both carotid arteries and a jugular vein were isolated and ligated anteriorly. A Millar pressure catheter was introduced into the left carotid artery to measure systolic arterial pressure (SAP), diastolic pressure (DAP), mean arterial pressure (MAP) and heart rate (HR). Another Millar pressure catheter was introduced into the left ventricular chamber through the right carotid artery to measure left ventricular systolic pressure (LVSP) and left ventricular end diastolic pressure (LVEDP).

A cannula for test compound administration was placed into a jugular vein. A lead II electrocardiogram was recorded via needle electrodes placed in the right arm, left leg and chest and PR and QRS intervals were reported.

After rats were surgically instrumented, hemodynamic and electrocardiographic monitoring was initiated. Individual animals were deemed acceptable for use in the study if they exhibited acceptable hemodynamic parameters during the approximate 15 minute equilibration period. Animals were given five minute infusions of either englerin A or englerin B with 10 minutes between each escalating dose. The duration of the experimental testing was approximately 60 minutes.

At the end of each infusion period, approximately 0.5 ml of blood was withdrawn from the vena cava into a pre-chilled collection tube containing 2.5 ml of 90% ACN (acetonitrile):10% water. Samples were mixed by inverting several times, separated into two aliquots of 1.5 ml each and immediately placed in a freezer set to maintain approximately -20°C. One set of samples was used to measure englerin A and englerin B levels by LC-MS/MS as described above.

### Ethics statements for animal experiments

All activities involving laboratory animals were carried out in strict accordance with federal, state, local and institutional guidelines governing the use of laboratory animals in research. All protocols in this study were approved by the Novartis Institutes for Biomedical Research Institutional Animal Care and Use Committee and were designed to maintain animal welfare standards.

Animal experiments at Cordyamics were carried out and approved by the Animal Care Committee of the University of Illinois-Chicago, UIC Public Health Service Letter of Assurance: A-3460.01.

All surgery was performed under ketamine/xylazine anesthesia, and all efforts were made to minimize suffering.

## Results

### TRPC4 expression correlates with sensitivity to englerin A

**Fig 2:**
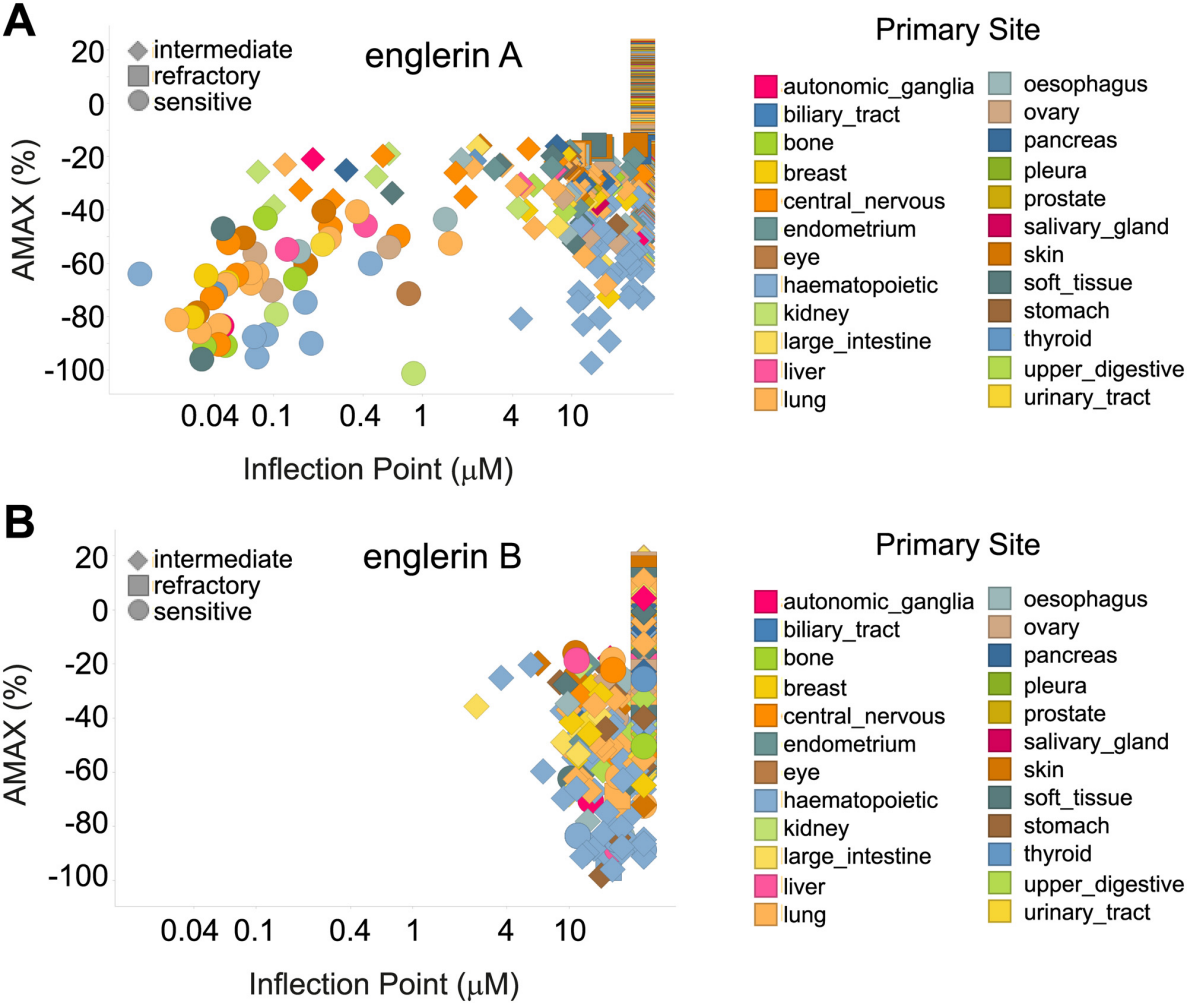
Englerin A affects proliferation of a subset of cancer cell lines across many cell lineages while englerin B is inactive. (**A**) Scatterplot of englerin A cell line profiling experiment. (**B**) Scatterplot of englerin B cell line profiling experiment. Each point represents effect of englerin A or B on growth of a single tumor cell line. Y-axis indicates maximal effect on growth and X-axis indicates potency. Tumor cell line lineage is indicated by color and the legend is in the figure. Englerin A sensitive (circles), englerin A refractory (squares) and englerin A intermediate (diamonds) cell line calls are indicated.

The effect of englerin A and englerin B (Fig 1) on proliferation of 524 cancer cell lines from the cancer cell line encyclopedia (CCLE)[13] was measured in a large scale cell profiling experiment (Fig 2). The scatter plot for englerin A shows that the compound is not generally anti-proliferative but potently blocks growth in a subset of cancer cell lines (Fig 2A). Englerin A sensitive cell lines occur in many different lineages (Table 1) suggesting that a genetic lesion or specific gene/pathway expression may be responsible for sensitivity to englerin A. Englerin B differs from englerin A only by the loss of a glycolate side chain (Fig 1). The englerin B scatter plot (Fig 2B) indicates no cancer cell lines have their growth potently inhibited by this molecule, making englerin B an ideal negative control for englerin A.

**Table 1:** Englerin A sensitivity calls across different tumor cell lineages.

Primary Site	Sensitive	Refractory	Intermediate
autonomic_ganglia	1	6	4
biliary_tract	0	1	0
bone	4	1	2
breast	3	21	13
central_nervous	6	9	12
endometrium	0	8	8
eye	1	2	0
haematopoietic	7	27	73
kidney	2	5	7
large_intestine	0	15	11
liver	2	2	6
lung	10	29	47
oesophagus	2	2	12
ovary	3	14	13
pancreas	0	11	7
pleura	0	1	3
prostate	0	0	3
salivary_gland	0	0	1
skin	4	30	11
soft_tissue	2	6	8
stomach	0	6	11
thyroid	1	0	1
upper_digestive	0	2	5
urinary_tract	1	4	3

**Fig 3:**
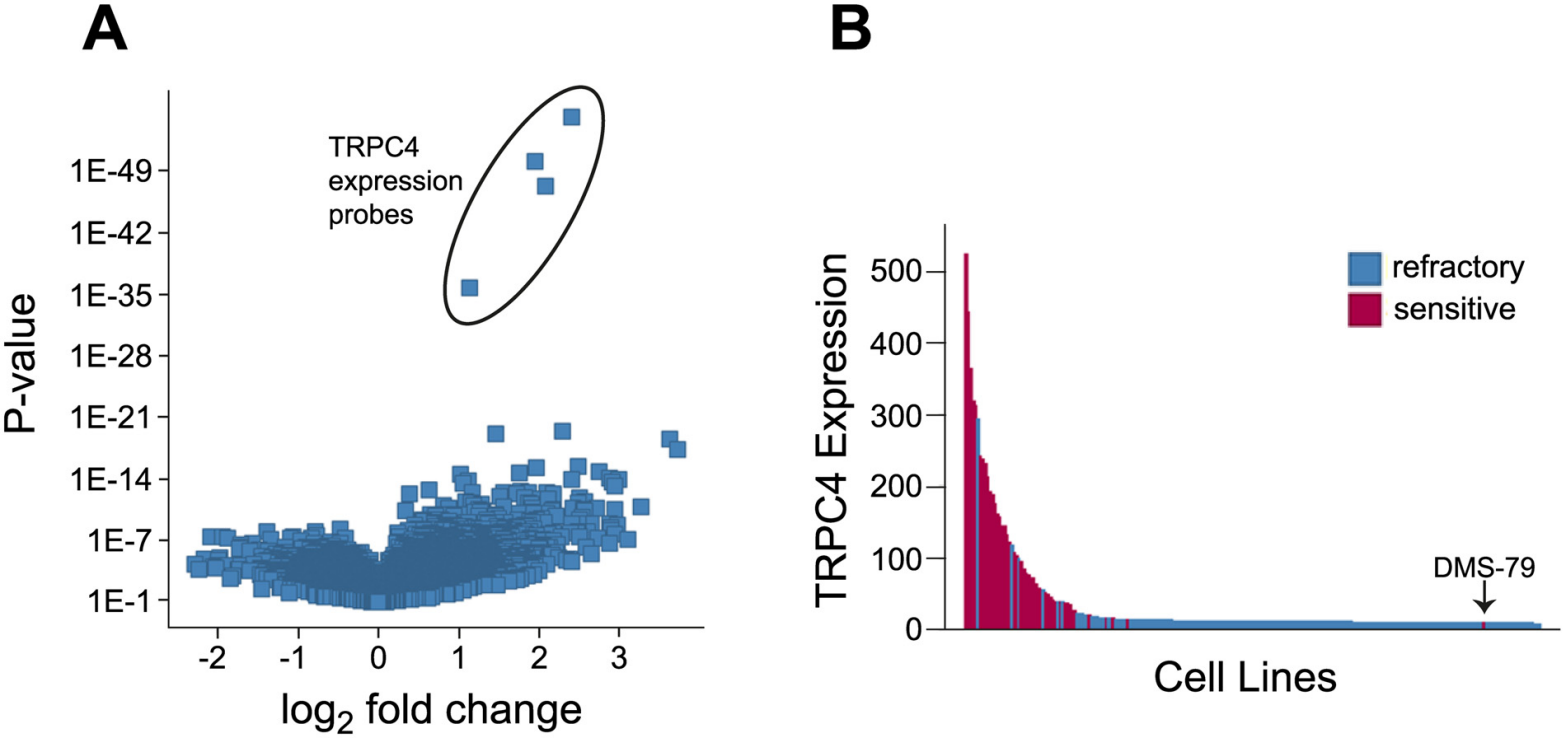
Sensitivity to englerin A induced growth inhibition correlates with expression of TRPC4. (**A**) Volcano plot of gene expression features. X- axis indicates fold change of feature in sensitive versus refractory cell lines and Y-axis indicates statistical significance. (**B**) Waterfall plot showing TRPC4 expression levels (affymetrix microarray units) in englerin A sensitive (red) and refractory (blue) tumor cell lines.

Cellular features of the englerin A-sensitive cell lines, including known mutations, gene expression levels, gene copy number and pathway activation were compared to features of lineage matched groups of englerin A resistant cell lines. The volcano plot of gene expression features (Fig 3A) shows that four Affymetrix probe sets stand out as most significantly associated with englerin A sensitivity. These probes all belong to the same gene, TRPC4. A waterfall plot of TRPC4 expression in englerin A sensitive and refractory cell lines indicates that the majority of sensitive cell lines have higher levels of TRPC4 mRNA expression than do the refractory cell lines (Fig 3B). Taken together, the data above shows that TRPC4 expression is the feature that best correlates with englerin A sensitivity and suggested the simple hypothesis that TRPC4 is the efficacy target for englerin A.

### TRPC4 is necessary and sufficient for cell proliferation effects of englerin A

**Fig 4:**
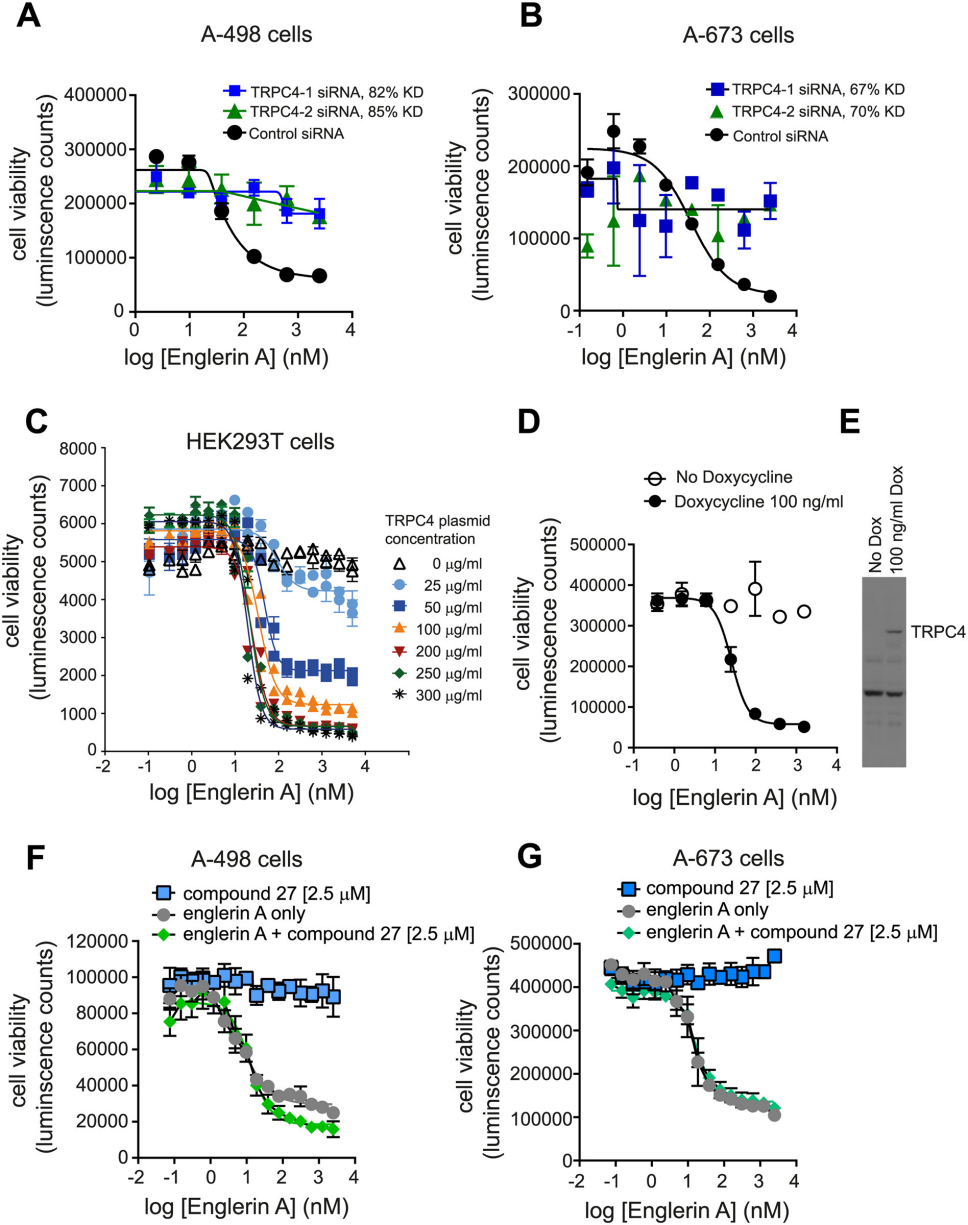
TRPC4 expression is necessary and sufficient for cell proliferation effects of englerin A. **(A**) Effect of TRPC4 siRNA knockdown on viability of A-498 cells in the presence of englerin A. An siRNA targeting luciferase was used as a control (mean+/- S.E.M.) Percent reduction of the TRPC4 mRNA levels are indicated in the legend, KD stands for knockdown. TRPC4 mRNA levels were normalized to peptidyl prolyl isomerase A (PPIA) mRNA levels. (**B**) Effect of TRPC4 siRNA knockdown on viability of A-673 cells in the presence of englerin A (mean +/- S.E.M.) (**C**) Effect of overexpression of TRPC4 by transient transfection on viability of HEK293T cells in the presence of englerin A. TRPC4 expression vector concentrations are indicated by different shapes (**D**) Effect of TRPC4 expression on cell viability in the presence of an englerin A in HEK293T cells engineered to express TRPC4 under control of a Doxycycline (Dox) regulated promoter (mean +/- standard deviation). 100 ng/ml Dox (black circles), 0 ng/ml Dox (open circles). (**E**) Western blot visualizing the levels of TRPC4 in the presence or absence of 100 ng/ml Dox. (**F**) Effect of PKCtheta inhibitor compound 27 on response to englerin A in A-498 cells. (**G**) Effect of PKCtheta inhibitor compound 27 on response to englerin A in A-673 cells.

Englerin A sensitive A-498 renal cell carcinoma cells were transfected with TRPC4 siRNAs to determine whether TRPC4 mRNA knockdown affects the cell’s response to englerin A. Two different siRNAs reduced TRPC4 mRNA levels by over 80% relative to a non-targeting control siRNA (Fig 4A). The A-498 cells treated with the control siRNA showed the characteristic concentration-dependent growth suppression in the presence of englerin A. In contrast, englerin A had little effect on the growth of cells treated with TRPC4 siRNAs (Fig 4A). Similar results were seen with englerin A-sensitive A-673 cells (Fig 4B). These data indicate that TRPC4 is necessary for englerin A induced growth suppression. They also suggest that englerin A is not a TRPC4 antagonist, since RNAi knockdown of the TRPC4 channel did not mimic the growth effect of englerin A.

The cell line, HEK293T, can be easily transfected and its growth is not affected by englerin A. To determine whether TRPC4 expression alone is sufficient to render cells sensitive to englerin A, TRPC4 was overexpressed in HEK293T cells by transient transfection (Fig 4C). The growth of mock transfected HEK293T cells was not affected by englerin A. As the concentration of TRPC4 plasmid was increased, the cells became more sensitive to growth inhibition by englerin A, up to an IC_50_ value of approximately 20 nM. Since transient transfection can lead to massive protein overexpression, a stable HEK293T cell line was constructed expressing TRPC4 under control of a doxycycline-inducible promoter. In these cells, a moderate level of TRPC4 protein was detectable on a Western blot after growth in doxycycline (Fig 4E). In the absence of doxycycline, the TRPC4-inducible HEK293T cell line was not sensitive to growth inhibition by englerin A (Fig 4D). After TRPC4 was induced, growth was inhibited with an IC_50_ of approximately 28 nM (Fig 4D). These data show that expression of TRPC4 is sufficient to confer sensitivity to englerin A in an otherwise englerin A resistant cell line.

It has been reported that englerin A blocks growth of sensitive cell lines by direct activation of the PKCtheta [6]. The levels of PKCtheta in englerin A sensitive A-498 and A-673 cells were so low they could not be detected by qRT-PCR (data not shown). This may not be surprising as PKCtheta is reported to be expressed predominantly in T lymphocytes[19]. To assess if any residual PKCtheta affects response of cells to englerin A, a published PKCtheta inhibitor called compound 27 was used[20]. The presence of 2.5 μM compound 27 has no effect on A-498 and A-673 cell proliferation on its own and does not alter the ability of englerin A to block cell growth (Fig 4F and 4G) In the cell lines tested, PKCtheta is unlikely to be the efficacy target of englerin A.

### TRPC5 expression can also confer sensitivity to englerin A

**Fig 5:**
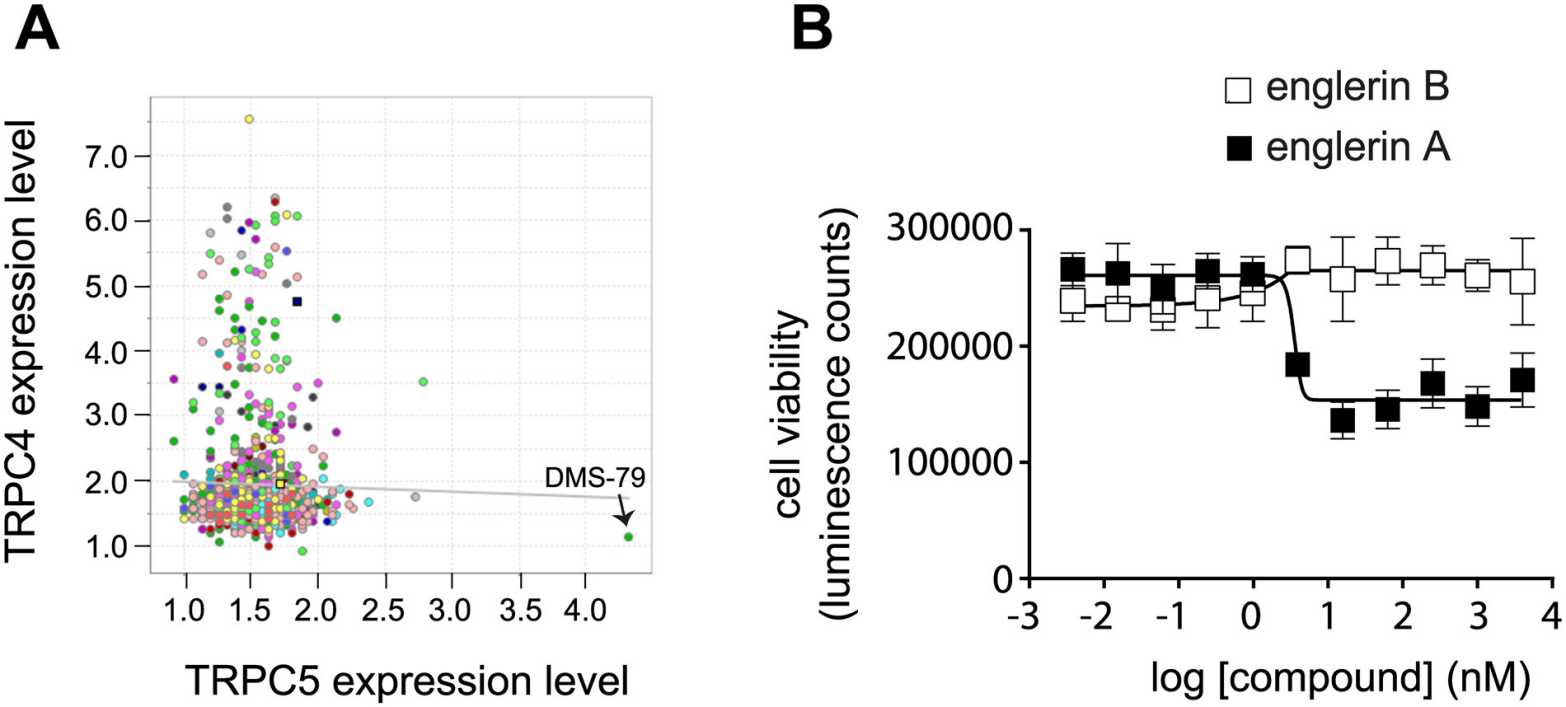
Overexpression of TRPC5 can also confer sensitivity to englerin A. (**A**) Expression of TRPC4 and TRPC5 in the cell lines from the CLiP experiment. Each circle represents a single cell line and expression levels were measured by microarray (TRPC4 probe 220818_s_at, TRPC5 probe 220552_at). The circle representing the DMS-79 cell line is indicated. (**B**) The effect of englerin A on cell viability in HEK293T cells transiently transfected with a vector expressing TRPC5 (mean +/- standard deviation).

In the cell profiling data, the DMS-79 small cell lung cancer cell line was a striking outlier because it was sensitive to englerin A but had low levels of TRPC4 expression (Fig 3B). A plot of the TRPC4 and TRPC5 expression data for the profiled cell lines indicates that DMS-79 cells express very high levels of TRPC5 (Fig 5A). Among the TRPC channels, TRPC5 is most closely related to TRPC4 sharing over 65% identity[21]. Overexpression of TRPC5 in HEK293T cells sensitized the cells to growth inhibition by englerin A (Fig 5B). These data suggest that cell lines can also be englerin A sensitive by expressing TRPC5.

### Englerin A is an agonist of the TRPC4 and TRPC5 cation channels

**Fig 6:**
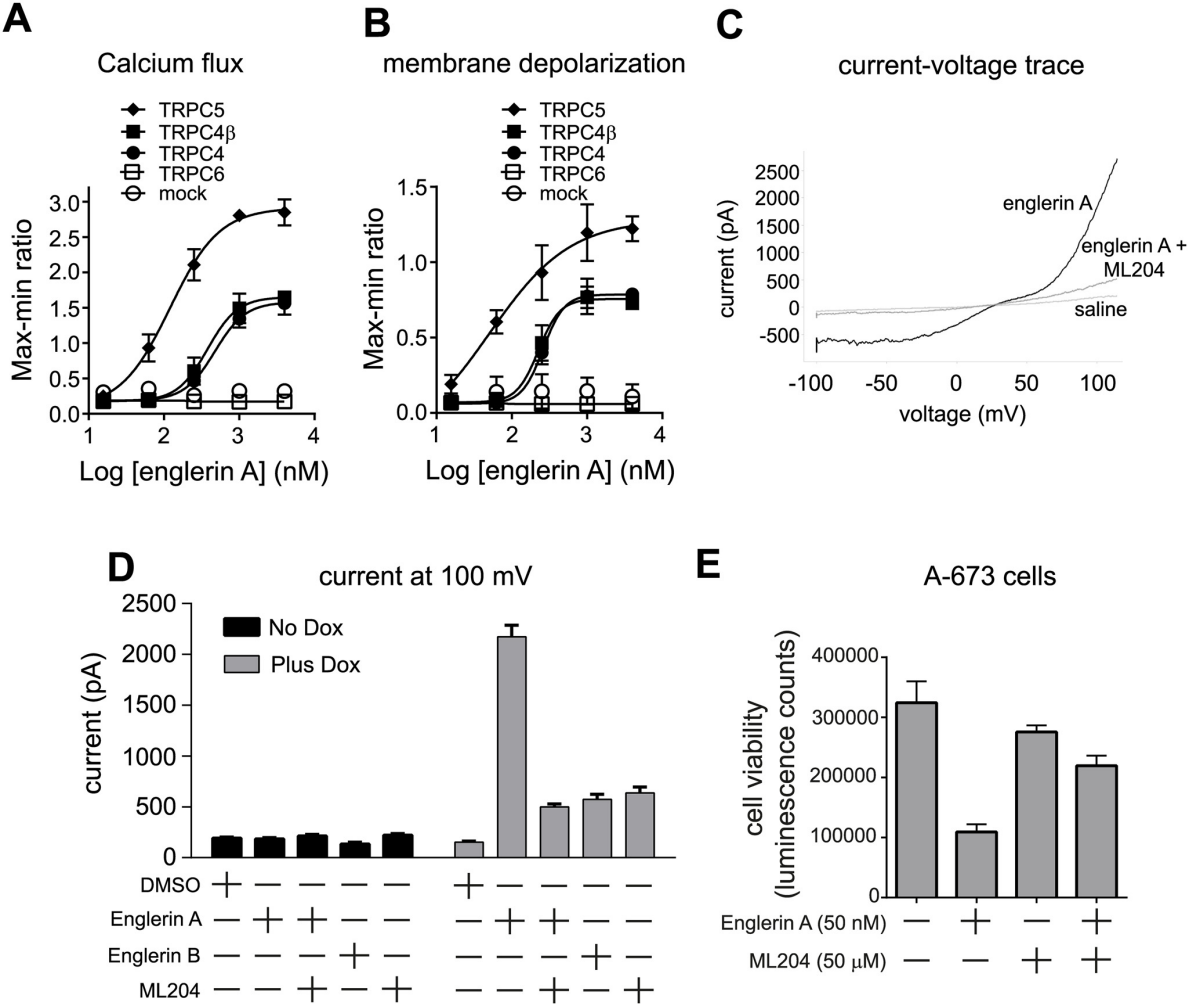
Englerin A agonizes the TRPC4/C5 ion channels and channel activation is needed for cell growth inhibition. (**A**) Calcium flux stimulated by englerin A in HEK293T cells overexpressing different TRPC proteins (mean +/- standard deviation): TRPC5 (closed diamonds), TRPC4beta (closed squares), TRPC4 (closed circles), TRPC6 (open squares), mock transfected cells (open circles). (**B**) Membrane depolarization stimulated by englerin A in HEK293T cells overexpressing different TRPC proteins (mean +/- standard deviation), markers as above. (**C**) TRPC4 current evoked by stimulation of 5 μM Englerin A, saline, or 5 μM Englerin A + 10 μM ML204 in 293T cells with Doxycyline-induced TRPC4. Currents were elicited by 200 ms voltage ramps from -100 to +100 mV, applied every 10 s from holding potential of 0 mV. (**D**) Summary of englerin A, englerin-B and ML-204 activity on membrane currents (mean +/- S.E.M.) (**E**) A-673 cell viability in the presence or absence of 50 nM englerin A and/or 50 μM ML204, a TRPC4/C5 channel blocker (mean +/- standard deviation).

TRPC4 and TRPC5 are reported to be non-selective sodium and calcium ion channels[22, 23]. The ability of englerin A to induce calcium influx in TRPC4/C5 expressing cells was assessed using a calcium binding dye assay. Englerin A caused a dose-dependent increase in intracellular calcium in HEK293T cells expressing TRPC4 or TRPC5, but not in mock transfected cells or in cells expressing TRPC6 (Fig 6A). A dye detecting membrane depolarization shows a similar profile (Fig 6B). TRPC4beta is a splice variant of TRPC4(22) which is also activated by englerin A leading to an increase in intracellular calcium in TRPC4beta overexpressing cells. These data indicate that englerin A activates the TRPC4/C5 channels resulting in an increase in intracellular calcium and membrane depolarization.

Electrophysiology experiments were performed on HEK293T cells with Dox-inducible expression of TRPC4. Addition of 5 μM englerin A to TRPC4 expressing cells stimulated a current across the membrane in electrophysiology patch clamp experiments using a voltage ramp protocol (Fig 6C). There was a strong outward current at 100 mV (1514±320 pA) and an inward current at -100 mV (-310±88 pA) which remained stable during the two minutes of englerin A exposure. The voltage-current relationship is doubly rectifying which is characteristic of TRPC4/C5 channels [24]. Both the inward and outward currents were inhibited by addition of the TRPC4/C5 channel inhibitor ML204. Englerin A induced no current in HEK293T cells not induced to express TRPC4 (Fig 6D). These data suggest that the englerin A-induced current is TRPC4 mediated.

While TRPC4/C5 expression is important for growth inhibition by englerin A, it is not clear whether channel activity is important for growth inhibition or whether some unidentified function of TRPC4/C5 is responsible. The englerin B analog, which is inactive in growth inhibition, did not induce current in TRPC4 expressing HEK293T cells (Fig 6D), suggesting a link between activity on TRPC4 channels and the ability to inhibit cell proliferation. To further address this question, a TRPC4/C5 channel blocker, ML204 (23), was tested for its ability to rescue growth inhibition by englerin A. A-673 cells treated with 50 nM englerin A show a moderate reduction in growth relative to control, and this growth effect could be partially rescued by addition of 50 μM ML204 (Fig 6E). This experiment was limited by the fact that A-673 cell growth is impaired by ML204 alone at doses higher than 50 μM. However, these data suggest that TRPC4 channel activity is responsible for englerin A mediated cell growth inhibition.

### Englerin A is selective for TRP family of ion channels

To better understand the selectivity of englerin A for TRPC4/C5, englerin A was tested against a panel of TRP family channel assays. Englerin A was shown to weakly inhibit TRPA1, TRPV3, TRPV4, and TRPM8 with an IC_50_ in the 3–4 μM range (Table 2). It was not found to activate any of these TRP channels or to inhibit TRPC6. Englerin A had no activity against non-TRP channels in functional assays (Table 2) or in binding assays for the nicotinic, NMDA, and 5HT3 channels (data not shown).

**Table 2:** Englerin A selectivity against TRP family and non-TRP family ion channels.

TRPchannel Assay	Mode	EC-50(μM)	Non-TRPchannel assay	Mode	EC-50(μM)
TRPA1	agonist	>30	ENaC	antagonist	>30
TRPA1	antagonist	2.62	GABA	antagonist	>30
TRPV3	agonist	>30	CFTR	antagonist	>30
TRPV3	antagonist	2.84	Nav1.5	antagonist	>50
TRPV4	agonist	>30	Cav1.2	antagonist	>50
TRPV4	antagonist	3.91	KcnQ1.MinK	antagonist	>50
TRPM8	agonist	>30			
TRPM8	antagonist	2.17			
TRPC6	antagonist	>30			

The interaction of englerin A with known receptors, transporters, nuclear receptors, kinases and enzymes were assessed in a panel of 162 binding and functional assays. All assay results were IC_50_ (or Ki) > 10 μM except human pregnane X (PXR), cannabinoid CB2, estrogen Erβ and serotonin 5 HT5A receptors. Englerin A had modest effects on these targets as it activated PXR receptors with an EC_50_ of 1.5 μM and activated remaining receptors only 50–60% at 10 μM. These data suggest that englerin A can interact with some TRP channels and does not interact with the set of non-TRP channels tested or other common pharmaceutical target classes.

### Englerin A is lethal to rodents

**Fig 7:**
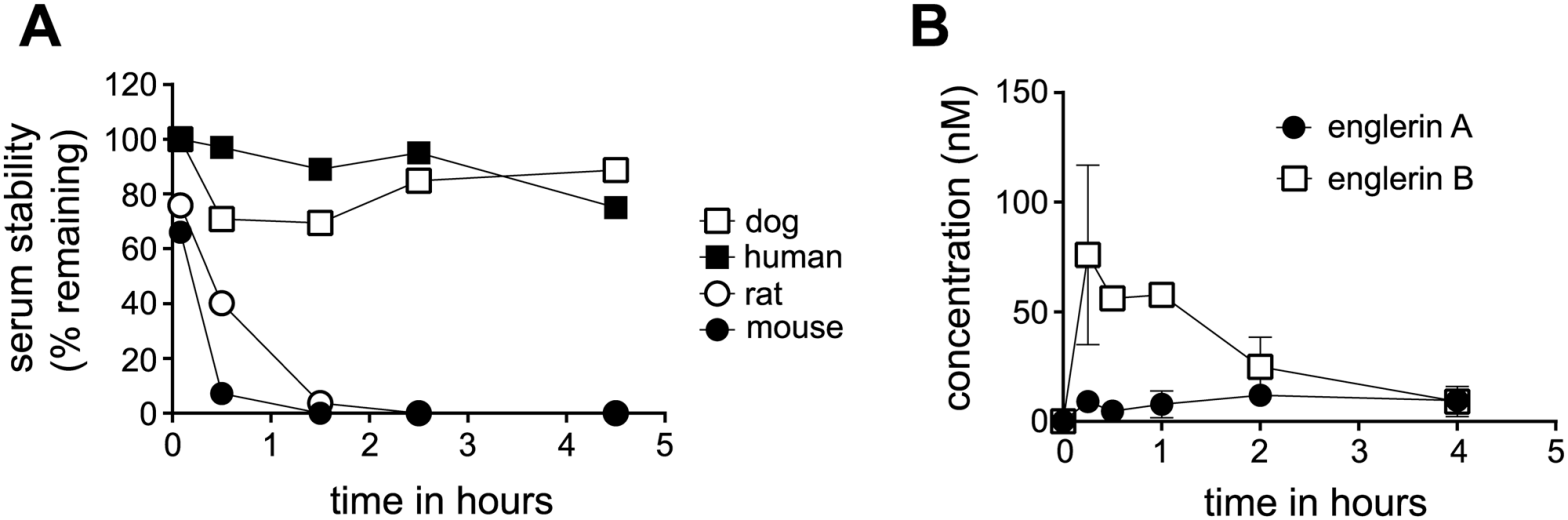
Serum stability and pharmacokinetics of englerin A. (**A**) Levels of englerin A detected after incubation in serum from dog (open square), human (black square), rat (open circle), and mouse (black circle). (**B**) Pharmacokinetics of englerin A in rats after a single oral dose of 5 mg/kg (mean +/- S.E.M.), englerin A (black circle) and englerin B (open square).

In preparation for *in vivo* studies, the plasma stability of englerin A was studied. Englerin A was stable in plasma from dogs and humans but was unstable in plasma from rats and mice (Fig 7A). There is virtually no englerin A remaining after 90 minutes incubation in rat or mouse plasma. This plasma instability suggested that it may be difficult to achieve significant exposure of englerin A in rodents. To determine the exposure and pharmacokinetic (PK) parameters in rat, an oral dose of englerin A (5 mg/kg) was administered and well tolerated (Table 3). However the levels of englerin A detected in blood never exceeded 12 nM (Fig 7B). Interestingly, higher blood levels of englerin B were observed (>50 nM) after dosing englerin A, suggesting that the rapid metabolism or plasma instability seen *in vitro* is recapitulated *in vivo* (Fig 7B). Conversion of englerin A into englerin B can occur through cleavage of the glycolate group. It is known that rodents have high levels of plasma esterases [25] which may explain the instability of englerin A in plasma from rats and mice.

**Table 3:** Summary of pharmacokinetic experiments performed with englerin A.

Species strain	Dosing route	Dose	Tolerability	Exposure
Rat Sprague Dawley	PO oral	5 mg/kg	Tolerated	<12 nM
Rat Sprague Dawley	IV intravenous	1 mg/kg	Rat died immediately after administration	Not determined
Rat Sprague Dawley	IP intraperitanial	5 mg/kg	Two rats died after rapid adverse effects (<15 minutes)	Not determined
Mouse C57BL/6	SC subcutaneous	1.5 mg/kg	Tolerated	Below level of detection (<113 nM)

To further investigate the PK properties of englerin A, rats were given a single intravenous (IV) dose of englerin A at 1 mg/kg. Surprisingly, the dosed animal died immediately after administration of the englerin A (Table 3). Because englerin A is rapidly converted to englerin B, it was not immediately clear if englerin A or the TRPC4 inactive englerin B was the toxic species in this experiment. In xenograft experiments published in the literature, nude mice tolerated 5 mg/kg doses of englerin A given intraperitoneally (IP) [6]. Use of the reported formulation (50% DMSO) was not possible because DMSO is not on the list of Novartis-approved excipients, however IP dosing of rats with 5 mg/kg of englerin A was done using a different formulation (5% ethanol, 10% PEG300, 25% cremaphor EL (20% v/v), and PBS). The dosed rats died within 15 minutes and no blood samples were collected (Table 3). The tolerability of IP dosing of englerin A was also assessed in nude mice, and no dose from 1 to 10 mg/kg was tolerated (Table 4).

**Table 4:** Summary of tolerability experiments done in nude mice on englerin A.

Dosing route	Dose	Comments
PO oral	1,3,5 &10 mg/kg	Tolerated at all doses tested
IP intraperitaneal	1,3,5 &10 mg/kg	Not tolerated at all doses tested
SC subcutaneous	5 mg/kg	Labored breathing, euthanized at 8 hours
	3 mg/kg	Hypoactive- recovered by 8 hours
	1.5 mg/kg	Mild injection site swelling
	0.5 mg/kg	Mild injection site swelling
	0.002 to 0.1 mg/kg	No clinical signs

Subcutaneous (SC) dosing can lead to sustained low exposure of compounds and could be a dosing route suitable for englerin A [26]. In nude mice, animals tolerated SC dosing of englerin A to 1.5 mg/kg and only mild injection site reactions were observed at 0.5 and 1.5 mg/kg (Table 4). At 5 mg/kg the mice were observed to have labored breathing and had to be euthanized after 8 hours. In a C57BL/6 mouse PK experiment with animals dosed subcutaneously at 1.5 mg/kg, the levels of englerin A were below the levels of detection (<113 nM for that experiment) (Table 3).

**Fig 8:**
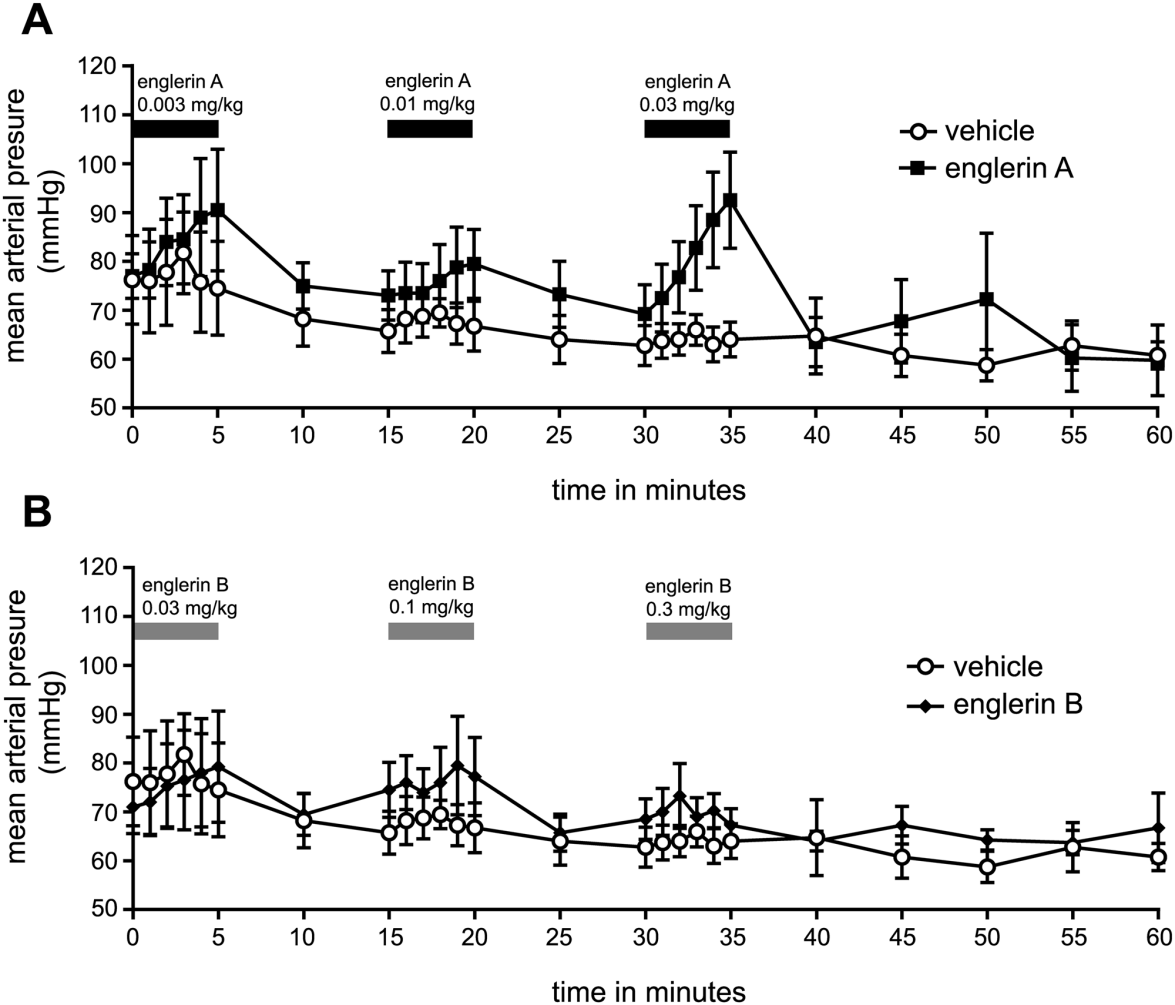
Effects of englerin A and englerin B on mean arterial pressure in anesthetized rats. (**A**) Mean arterial blood pressure of anesthetized rats dosed with englerin A (mean +/- S.E.M.). (**B**) Mean arterial blood pressure of anesthetized rats dosed with englerin B (mean +/- S.E.M.).

To more carefully investigate the toxicity of englerin A and englerin B, these molecules were intravenously dosed into anesthetized rats and cardiovascular parameters were monitored. At a dose of 0.03 mg/kg englerin A caused a steady increase in mean arterial pressure (MAP) in the rats (Fig 8A). This effect on blood pressure is observed at concentrations of englerin A from 30–63 nM in systemic circulation (Table 5). No significant effect on MAP was seen at doses of 0.01 mg/kg or 0.003 mg/kg of englerin A and no apparent effects on other cardiovascular parameters including cardiac contractility were observed. In anesthetized rat experiments, two of the animals died following administration of englerin A infused with 0.03 mg/kg and 0.1 mg/kg. In these animals a rapid rise in blood pressure was followed by a sudden reduction in blood pressure. Englerin A reached levels of 132 nM and 544 nM in the systemic circulation of these animals (Table 5). In contrast, englerin B had no effect on mean arterial pressure or animal viability at doses up to 0.3 mg/kg (Fig 8B) which yielded systemic exposures of 362–539 nM of englerin B (Table 5). These data suggest first that englerin A and not englerin B is responsible for the observed toxicity. Second, englerin A is lethal in rats if concentrations in the systemic circulation reach approximately 100 nM.

**Table 5:** Englerin A and B plasma levels and effects on animal health in anesthetized rat experiments.

Compound	Dose	Experimental animal #	Compound concentration in blood	Outcome
Englerin A	0.03 mg/kg	15	30 nM	Increase MAP
	0.03 mg/kg	8	63 nM	Increase MAP
	0.03 mg/kg	7	132 nM	Animal died
	0.1 mg/kg	8	544 nM	Animal died
Englerin B	0.3 mg/kg	9	362 nM	No effect
	0.3 mg/kg	10	462 nM	No effect
	0.3 mg/kg	11	442 nM	No effect
	0.3 mg/kg	12	539 nM	No effect

## Discussion

In this work, we identified TRPC4 and TRPC5 as the cellular efficacy targets of englerin A which lead to growth inhibition in cancer cell lines. Large scale cell line profiling (CLiP) was used to generate a target hypothesis which was confirmed in follow up genetic and cell physiology experiments. Expression of TRPC4 in cancer cell lines was necessary and sufficient for englerin A mediated growth inhibition. Addition of englerin A activated calcium influx and membrane depolarization which was dependent on TRPC4 or TRPC5 expression. Electrophysiology experiments demonstrated that englerin A activates currents in TRPC4 expressing cells but not in cells with no TRPC4 expression. The activated currents have an I-V profile characteristic of TRPC4/C5 channels and the current can be blocked by ML204, a TRPC4/C5 inhibitor. ML204 can also rescue the growth effect of englerin A in A-673 cells indicating that the TRPC4 channel activity is required for inhibition of cell proliferation. Englerin A is the first low molecular weight compound identified which activates TRPC4 and TRPC5 channels. While this manuscript was being prepared, Akbulut et al. published that englerin A activates TRPC4/C5 channels[14].

Many factors contributed to the clarity with which the CLiP experiment pointed to TRPC4 as a target hypothesis. One factor is that a large number of well characterized cell lines were profiled. Increasing the number of cell lines tested in the future will continue to increase the power of this type of experiment. Importantly, the growth inhibition caused by englerin A occurred across many cell lineages which allowed lineage specific markers to be eliminated as target hypotheses. If englerin A had only affected RCC cells, then all unique features of RCC cells would have correlated with englerin A sensitivity and no clear target hypothesis would have emerged. Another important reason the sensitivity analysis on the cell line profiling data yields a validated result is that a single marker, TRPC4, robustly correlates with sensitivity. It is probably more common to have multiple sources of sensitivity and/or resistance, which would make it difficult to discern true signal from false positives. It is helpful that englerin A has a strong effect on cell proliferation, the phenotype tested and that a good proportion of the cell lines overexpressed that target. For example, TRPC5 was not identified as a target hypothesis because TRPC5 is rarely overexpressed in tumor cell lines (Fig 5*A*). This work shows that cell line profiling on its own can generate a strong compound target hypothesis.

There are several cell lines that have high expression of TRPC4 yet were not sensitive to englerin A (Fig 3*B*). Many of these cell lines express high mRNA levels of UDP-glucuronosyltransferases (UGTs), a class of enzymes that transfer glucuronic acid to small lipophilic molecules to aid in their clearance[27]. Englerin A is a substrate for UGT enzymes and cell lines with high UGT expression may become resistant to englerin A due to rapid englerin A clearance. Other exceptions might occur due to many reasons: high TRPC4 mRNA expression levels may not translate into high protein levels of TRPC4, unknown negative regulators may keep TRPC4 activity low and cells might have compensatory mechanisms to block membrane depolarization or secrete excess calcium. The presence of high TRPC4 expressing, englerin A refractory cell lines in CLiP highlights the difficulty of predicting compound sensitive cell lines using a single mRNA expression marker.

The mechanism by which englerin A activates the TRPC4/C5 channels is not understood. We favor a model in which englerin A binds directly to TRPC4 to open the channel although it is also possible that englerin A acts upstream of the channel to increase its activity. The fact that englerin A can inhibit other TRP family channel members suggests that englerin A interacts with a common feature of TRP channels (Table 2). Like all TRP channels, TRPC4 has N and C terminal cytoplasmic domains involved in signal transduction flanking six transmembrane domains[21, 28, 29]. Four TRPC4 proteins combine to form the channel and the pore is comprised of transmembrane segments 5–6 from each of the four TRPC4 proteins. Englerin A could bind TRPC4 like resinferatoxin binds to TRPV1, which occupies a hydrophobic pocket near the transmembrane domains and opens the lower gate of the channel[30, 31]. Englerin A might also covalently modify cysteine residues in TRPC4 in analogy to the way that environmental irritants covalently modify cysteine residues to activate TRPA1[32]. The covalent binding hypothesis is attractive because englerin A can theoretically react with cysteine thiol groups to transfer the glycolate moiety to the protein and in the process release englerin B. The glycolate group of englerin A is essential for its activity, as demonstrated by the lack of activity seen with englerin B. Experiments to test the covalent binding model did not yield conclusive results.

The mechanism(s) by which TRPC4 channel agonism leads to growth inhibition in tumor cell lines are not obvious. TRPC4 is a non-selective cation channel whose opening will allow both sodium and calcium flows into the cell[33]. The sodium flux is likely to be responsible for cell membrane depolarization. Cell proliferation requires spatially and temporally regulated increases in calcium levels[34]. It is possible that the activation of TRPC4 disrupts these calcium signals blocking proliferation in various phases of the cell cycle[35]. It is well known from the ion channel field that expression of active channels in cells can be incompatible with cell viability. It is also not clear why some tumor cell lines express TRPC4 at high levels. TRPC4 is located at chromosomal locus 13q13.1-q13.2[21], a region that is not commonly amplified in cancer[36]. An analysis of the promoter region of TRPC4 identified no binding sites for transcription factors commonly expressed in tumors. It is possible that increased levels of TRPC4 may potentiate a GPCR pathway that confers a proliferation or survival advantage to the tumor cell lines. Further work will be needed to answer these questions.

Englerin A was observed to be acutely toxic in rodents despite the fact that the molecule is highly unstable in rodent plasma. TRPC4 and TRPC5 are not essential for viability or development as both knockout animals reach adulthood[37, 38]. The knockout phenotype might mimic a channel inhibitor, however englerin A is channel agonist. Several pieces of evidence suggest the toxicity of englerin A is caused by on-target effects on TRPC4/C5 channels. First, the closely related englerin B, which is inactive against TRPC4/C5, does not show toxicity in rodents. Second, the concentrations at which englerin A activates a calcium flux in TRPC4 expressing cells (~250 nM) is very close to the concentrations at which englerin A was lethal in rodents (132–544 nM). Interestingly, englerin A appears most potent in cellular toxicity assays (lowest IC_*50*_ ~30 nM) which can probably explained by the fact that the growth effect experiments occur over a long period of time, 24 hours or longer, while the calcium flux experiments measure effects in seconds. Third, englerin A is a selective molecule which is inactive against many known targets classes, reducing the chance of off-target toxicity at such low concentrations. Definitive proof that TRPC4 or TRPC5 is required for englerin A toxicity will have to come from dosing englerin A in knockout mice or rats.

We suspect that the observed lethality of englerin A in rat is caused by leakage of the vasculature of the lung (pulmonary edema). The ability of thrombin to increase vascular permeability of the lung requires TRPC4[39]. Englerin A, as a TRPC4 agonist, may mimic the thrombin signal and cause uncontrolled vascular permeability. This hypothesis is consistent the observations that englerin A treated mice were gasping for breath and that fluid was observed in the tracheal tubes of rats which died after englerin A treatment. TRPC4 and TRPC5 are also expressed in many areas of the mouse and rat brain[40]. TRPC5 expression is limited to the brain, while TRPC4 expression is also found in the olfactory bulb, uterus, ovary, kidney, and in endothelial cells[37]. The modest increase in mean arterial pressure seen in the anesthetized rats may have occurred because of activation of TRPC4 expressed in the smooth muscles of the arteries directly leading to an increase in blood pressure[41].

In conclusion, englerin can activate TRPC4 and TRPC5 ion channels at nanomolar concentrations, and englerin A can inhibit growth of tumor cell lines which express high levels of TRPC4 or TRPC5. However, englerin A is extremely toxic in rodents despite being highly unstable in rodent serum. This toxicity of englerin A would likely be more pronounced in humans because englerin A is stable in human plasma. Since englerin A can be synthesized in the laboratory, it could be an interesting starting point for identifying novel modulators of other TRP channels, but in our opinion englerin A itself is a poor candidate for drug development.

## Supporting Information

S1 Table Effects of englerin A and englerin B on growth of 517 cancer cell lines. (XLSX)
